# PTIP Associated protein 1, PA1, Is an Independent Prognostic Factor for Lymphnode Negative Breast Cancer

**DOI:** 10.1371/journal.pone.0080552

**Published:** 2013-11-18

**Authors:** Takashi Takeshita, Mutsuko Yamamoto-Ibusuki, Yutaka Yamamoto, Yoko Omoto, Yumi Honda, Ken-ichi Iyama, Zhenhuan Zhang, Hirotaka Iwase

**Affiliations:** 1 Department of Breast and Endocrine Surgery, Graduate School of Medical Science, Kumamoto University, Kumamoto, Japan; 2 Department of Molecular-Targeting Therapy for Breast Cancer, Kumamoto University Hospital, Kumamoto, Japan; 3 Department of Surgical Pathology, Kumamoto University Hospital, Kumamoto, Japan; Rutgers - New Jersey Medical School, United States of America

## Abstract

Pax transactivation domain interacting protein (PTIP) associated protein 1, PA1, was a newly found protein participating in the modulation of transactivity of nuclear receptor super family members such as estrogen receptor (ER), androgen receptor (AR) and glucocorticoid receptor (GR). Breast cancer is one of the most life threatening diseases for women and has tight association with estrogen and ER. This study was performed to understand the function of PA1 in breast cancer. The expression of PA1 had been evaluated in a total of 344 primary invasive breast cancer samples and examined the relationship with clinical output, relapse free survival (RFS), breast cancer-specific survival (BCSS). PA1 expression was observed in both nucleus and cytoplasm, however, appeared mainly in nuclear. PA1 nuclear expression was correlated with postmenopausal (P = 0.0097), smaller tumor size (P = 0.0025), negative Ki67 (P = 0.02), positive AR (P = 0.049) and positive ERβ (P = 0.0020). Kaplan–Meier analysis demonstrated PA1 nuclear positive cases seemed to have a longer survival than negative ones for RFS (P = 0.023) but not for BCSS (P = 0.23). In the Cox hazards model, PA1 nuclear protein expression proved to be a significant prognostic univariate parameter for RFS (P = 0.03), but not for BCSS (P = 0.20). In addition, for those patients without lymphnode metastasis PA1 was found to be an independent prognostic factor for RFS (P = 0.025), which was verified by univariate and multivariate analyses. These investigations suggested PA1 expression could be a potential prognostic indicator for RFS in breast cancer.

## Introduction

Breast cancer is the most commonly diagnosed cancer and the second leading cause of cancer deaths among American women, and thus has been identified as a public health priority in the United States. The lifetime risk of developing breast cancer today is one in every eight women [1]. The incidence of breast cancer in Japanese women has doubled in all age groups over the past two decades and we have recently shown that this marked increase is mostly due to an increase in the estrogen receptor (ER)-positive subtype, especially in women aged 50 years or less [2]. Although ER plays a central role in the prognosis and endocrine therapy responsiveness prediction, ER as a transcription factor can be modulated by various cofactors and be cross interacted with other signal pathways [3-7], which indicates ER is not a perfect index for the treatment of breast cancer. Extensive searching for the modulation factors is of urgent importance for the endocrine therapy of breast cancer patients. Our work in this field with others is highly clinically relevant, and admired by both patients and clinicians, and led to the discovery that several cofactors, such as nuclear receptor corepressor 1 (NCOR1), histone deacetylase 1 (HDAC1), and histone deacetylase 6 (HDAC6), which have clinical importance to enhance the accuracy of prediction of endocrine therapy responsiveness and prognosis [8-10]. 

Unfortunately, tumor cells may develop resistance to endocrine therapy, which become a major obstacle limiting the success of breast cancer treatment. The complicated crosstalk, both genomic and nongenomic, between ER and growth factors was considered to be a crucial factor contributing to endocrine resistance. However, the progression of resistance to endocrine therapy supposes to be a progressive, step-wise procedure and the underlying mechanism remains unclear [8,11]. Crosstalk between the ER and human epidermal growth factor receptor 2 (HER2) pathways has been established as a pivot in both intrinsic and acquired resistance to endocrine agents. Nevertheless, the intrinsic and acquired resistance occurs in a significant proportion of patients and limits the efficacy of endocrine treatments. Several molecular mechanisms have been proposed to be responsible for endocrine resistance. Loss of ER expression, altered activity of ER co-regulators, deregulation of apoptosis and cell cycle signaling, and hyperactive receptor tyrosine kinase (RTK) and stress/cell kinase pathways can collectively orchestrate the development and sustenance of pharmacologic resistance to endocrine therapy [12]. Enormous efforts have been paid to the search for new ER transcription co-modulators. 

PA1, Pax transactivation domain interacting protein (PTIP) [13] from nuclear extracts prepared from HeLa cells, was first identified as a novel protein that were grown in the absence of DNA damage agent treatment and carried robust histone H3 lysine 4 (K4) methyltransferase activity [14]. PA1 (also designated as GAS) was found to interact with estrogen receptor-alpha (ERα), participate in both ER-regulated gene transcription and estrogen-stimulated G1/S cell-cycle transition, and interact only with steroid receptor co-activator 1 [15]. Our most recent study demonstrated that PA1 is a new competitive decelerator of glucocorticoid receptor (GR) transactivation and can act at more than one molecularly defined step in a manner that depends upon the specific gene and also inhibit androgen receptor (AR) transactivity [16].

The extensive role of PA1 in the modulation of steroid receptor functions, especially in ER, prompted us to investigate its role in clinical breast cancer tissues to explore its correlation with clinicopathological parameters of breast cancer patients. This work will benefit not only thousands of breast cancer patients, but will also expand the value of this cofactor PA1 in other malignant tumors. To our knowledge this is the first report of quantitative detection of PA1 protein expression in breast cancer and analysis of its correlation with clinicopathological parameters and prognosis. 

## Materials and Methods

### Ethics Statement

This retrospective study was conducted in accordance with the Helsinki Declaration and approved by ethics review board of Kumamoto University Graduate School of Medical Sciences. All patients signed informed consent forms.

### Patients and tumor samples

A total of 344 consecutive female with invasive breast carcinoma who were treated at Kumamoto University Hospital between 2001 and 2009, were enrolled in this protocol. The median age of the patients was 58 years of age (range, 21 - 93 years of age). Adjuvant and neoadjuvant treatment was administered in accordance with the recommendations of the St. Gallen international expert consensus on the primary therapy of early breast cancer [17-19]. Neoadjuvant treatments were administered to 71 patients (58 for chemotherapy which was 5FU 500 mg/m^2^, epirubicin 75 or 100 mg/m^2^, and cyclophosphamide 500 mg/m^2^ (FEC) followed by triweekly docetaxel 75 mg/m^2^ (DOC) or doxorubicin 60 mg/m^2^ and cyclophosphamide 500 mg/m^2^ (AC) followed by weekly paclitaxel 80 mg/m^2^ (PTX), and 13 for hormonal therapy which was aromatase inhibitors). For adjuvant therapy, a total of 180 (52.3%) of 344 patients were treated with hormonal therapy, 36 (10.4%) with chemotherapy which was FEC with or without (w/o) DOC or AC w/o PTX, 81 (23.5%) patients were treated with both hormonal and chemo-therapy, respectively. In addition, 18 patients were treated with targeted therapy using trastuzumab simultaneously. No therapy was administered to 35 patients, and no detailed information on therapy could be obtained for 12 patients. When tumor recurs, patients with hormone receptor-positive tumors and non-visceral metastases were treated with endocrine therapy, such as antiestrogens, aromatase inhibitors, and medroxyprogesterone acetate. For patients with Her2 overexpressed tumors, tastuzumab was applied. Other patients were treated with chemotherapy such as anthracycline containing regimens, taxanes, capecitabine, and vinorelbine. Patients were periodically examined at the Kumamoto University Hospital or affiliated hospitals. The patients were observed every 3 months for 5 years and every 1 year thereafter. Recurrence was defined when positive spots were found by physical examination and/or by imaging diagnosis during follow-up period. The median follow-up period was 66.5 months (range, 0.23 - 145 months). 

### Immunohistochemistry

All archival formalin-fixed, paraffin-embedded breast tumor specimens were cut into 4-μm sections, which were used for the present histopathological and immunohistochemical investigations. Immunohistochemistry for ERα, progesterone receptor (PgR), estrogen receptor β (ERβ), AR, and PA1 was performed as follows: The sections were deparaffinized, heated 60 min in citrate buffer (pH7) at 100 °C, for antigen retrieval and incubated for 10 min in distilled water containing 3% hydrogen peroxide. We used the rabbit polyclonal antibody against PA1 (#A301-978A, 1:3000; BETHYL laboratories.inc, Texas, USA) at 4 °C overnight. A rabbit polyclonal antibody was also used for the detection of Her2 (1:200; Dako Japan, Tokyo, Japan), and a mouse monoclonal antibodies was used for ERα (1D5, 1:50; Dako Japan), PgR (PgR636, 1:800; Dako Japan), ERβ (PPG5/10, 1:30, DAKO), AR (AR-318, 1:100, Leica), and Ki67 (MIB-1, 1:50; Dako Japan). Expression using these antibodies was determined by the Histofine Simple stain MAX-PO^®^ (Nichirei, Tokyo, Japan) method [20,21] for ERα, PgR and ERβ, and the VECTASTAIN Elite Avidin-Biotin Complex (ABC) (Vector Laboratories, CA, US) method for AR, respectively. The VECTASTAIN^®^ Elite ABC system has been done as the manufactures’ protocol. As a negative control, parallel sections were immunostained without exposure to primary antibodies. No immunoreactivity was observed in these sections.

### Immunohistochemical evaluations

This study was reported according to the Reporting Recommendations for Tumor Marker Prognostic Studies (REMARK) criteria [22]. PA1 expression was scored according to the respective different staining patterns. Nuclear staining and cytoplasmic staining were independently scored by Histo-Score (HS). The HS represented a product of the each staining intensity (from 0 negative, 1 weak, 2 moderate, to 3 strong), and the each percentage of positive cells (0-100%) with a maximum HS of 300. We counted approximately 100 cancer cells in five randomly chosen microscopic fields. Both nuclear and cytoplasmic PA1 HS was analyzed as a dichotomous variable using quartile values as cut-off points; negative ≤ 25^th^ percentile of HS, weakly positive > 25^th^ and 50^th^ ≤ percentiles of HS, moderately positive > 50^th^ and 75^th^ ≤ percentiles of HS, and strongly positive > 75^th^ percentile of HS. Negative was compared with others (weakly, moderately, and strongly positive; > 25^th^ to >75^th^ percentiles). ERα and PgR were considered positive when there was ≥ 1% of nuclear staining [23]. We decided AR and ERβ status positivity as ≥ HS 10 of nuclear staining for AR and ≥70 points (the 25^th^ percentile) for ERβ, respectively. Her2 immunostaining was evaluated using the Herceptest (Dako), the membranous staining and its distribution (range, from 0 to 3+). Tumors with scores of 3+ ≤ or with a ≥ 2.2 - fold increase in Her2 gene amplification as determined by fluorescence in situ hybridization were considered to be positive for Her2 overexpression. Ki67 was scored as the percentage of nuclear staining cells out of all cancer cells in the invasive front of the tumor at 40 high-power field (Ki67 labeling index). Cancer cells have been evaluated by counting in average of 500 cells and we assumed 15% a cut-off level [24]. 

### Statistical analysis

The nonparametric Mann-Whitney U test and contingency analysis were adopted for statistical analysis of the associations between different PA1 in the nucleus/cytoplasm status and the clinicopathological characteristics of the patients. The Spearman rank correlation coefficient was used to assess the correlation among ERα, PgR, ERβ, AR, and PA1 protein expression. For relapse-free survival (RFS) and breast cancer-specific survival (BCSS), Kaplan–Meier method was used to estimate survival rates, and differences between survival curves were evaluated by the log-rank test. Cox’s proportional hazards model was used for the univariate and multivariate analysis of prognostic status. The P values < 0.05 were considered a significant result. All reported P values are two-sided, and confidence intervals (CIs) are at the 95% level. All analyses were performed by using JMP software version 10.0.1 for Windows (SAS institute Japan, Tokyo, Japan).

## Results

### PA1 staining pattern and staining positivity

**Figure 1:**
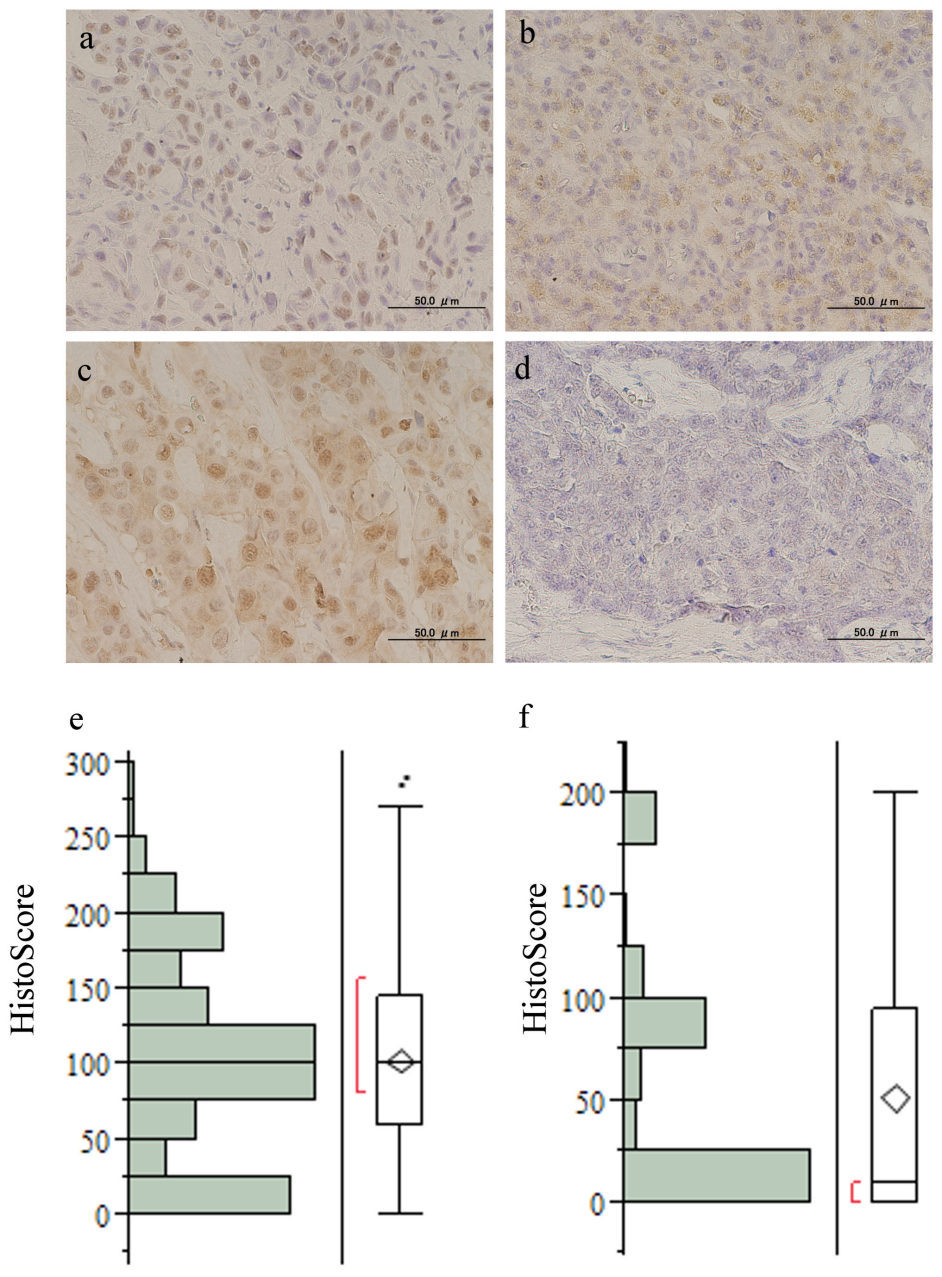
Representative microscopic views of PA1 staining with rabbit polyclonal antibody against PA1. a, nuclear staining; b, cytoplasmic staining; c, mixed nuclear and cytoplasmic staining; d, negative staining (original magnification × 400) e, histograms for Histo-Score (HS) of PA1-nuc and f, of PA1-cyto (mean; 51.4, S.D.; 62.4). Abbreviations: PA1-nuc, nuclear PA1 expression; PA1-cyto, cytoplasmic PA1 expression; S.D., Standard Deviation.

PA1 immunohistochemistry staining was done in 344 invasive breast cancer cases and was analyzed by HS (Figure 1). The mean HS was 102.8 (standard deviation (S.D.); 64.9) in nuclear PA1, 51.4 (S.D.; 62.4) in cytoplasmic PA1. Negative, weakly, moderately and strongly positive nuclear PA1 expression (PA1-nuc) were present in 91 (26.4%), 100 (29.0%), 68 (19.7%), and 85 (24.7%) cases, respectively. Cytoplasmic protein expression for PA1 (PA1-cyto) was negative in 167 (48.5%) cases, weakly positive in 6 (1.7%) cases, moderately positive in 92 (26.7%) cases and strongly positive in 79 (22.9%) cases. Pure PA1-nuc expression was present in 145 (42.1%), pure PA1-cyto in 9 (2.6%), and both nuclear and cytoplasmic staining (PA1-nuc/cyto) in 168 (48.8%) of 344 cases, respectively. 22 (6.3%) were not stained in either the nucleus or the cytoplasm.

### Association of PA1 protein expression with clinicopathological parameters

Because PA1-nuc and -cyto HS were analyzed as a dichotomous variable way so we demonstrate PA1-nuc and -cyto HS with clinicopathological factors and prognosis, respectively. The clinicopathological characteristics for the 344 cases analyzed in the present study are summarized in Table 1. The level of PA1 expression was observed to be significantly associated with several clinicopathological parameters. Higher PA1-nuc HS levels were indicated in the groups of patients with postmenopausal group (P = 0.0097), lower tumor size (P = 0.0025), negative Ki67 (P = 0.02), and positive AR (P = 0.049) and positive ERβ (P = 0.0020). No relationship could be found between PA1-nuc protein expression with lymph node metastasis (P = 0.21), histopathology (P = 0.54), nuclear grade (P = 0.45), hormone receptor status (for ERα, P = 0.21; for PgR, P = 0.63), Her2 status (P = 0.10), and subtypes (P = 0.2). Higher PA1-cyto protein expression had a remarkable relationship with postmenopausal group (P = 0.016) and positive ERα (P = 0.0038), but there was no correlation with any other clinical factors.

**Table 1:** Association of PA1 protein expression (Nuclear and Cytoplasmic) with clinicopathological parameters.

		PA1			
	Total	Nuclear		Cytoplasm	
Characteristic	(n = 344)	median (25%, 75%)	P value	median (25%, 75%)	P value
Age, y					
≤50	99	95 (56, 125)	NS	0 (0, 90)	NS
>50	245	100 (45, 122)		0 (0, 96)	
Menopausal state					
Pre-	95	91 (44,124)	0.0097*	0 (0,80)	0.016*
Post-	247	100 (70, 156)		20 (0,98)	
Tumor size					
≤ 2cm	188	105 (71, 159)	0.0025*	30 (0, 97)	NS
> 2 cm	155	95 (45, 126)		0 (0, 90)	
Axillary lymph nodes					
Negative	217	100 (70, 146)	NS	10 (0, 97)	NS
Positive	113	95 (40, 143)		30 (0, 93)	
Histopathology					
Ductal	302	100 (60, 145)	NS	10 (0, 95)	NS
Lobular	10	82 (9, 162)		20 (0, 99)	
Nuclear Grade					
1,2	266	100 (60, 148)	NS	20 (0, 98)	NS
3	74	98 (58, 135)		0 (0, 90)	
ERα					
Negative	62	97 (49, 135)	NS	0 (0, 81)	0.0038*
Positive	282	100 (60, 148)		30 (0, 96)	
PgR					
Negative	101	98 (55, 146)	NS	0 (0, 90)	NS
Positive	241	100 (60, 145)		0 (0, 98)	
HER2					
Negative	298	100 (60, 146)	NS	10 (0, 95)	NS
Positive	46	93 (30, 111)		10 (0, 91)	
Ki67 (MIB1 )					
<15 %	121	111 (60, 160)	0.02*	15 (0, 91)	NS
≥15 %	206	98 (60, 130)		10 (0, 95)	
AR					
Negative	45	98 (13, 135)	0.049*	0 (0, 90)	NS
Positive	293	100 (70, 148)		20 (0, 95)	
ERβ					
Negative	77	90 (14, 118)	0.0020*	0 (0, 90)	0.051
Positive	218	102 (84, 150)		40 (0, 98)	
Subtype					
HR+ ^a^/HER2-	264	100 (63, 148)	NS ^$^	22.5 (0, 95)	NS ^$^
HER2+	46	93 (30, 111)		10 (0, 91)	
HR-/ HER2-	34	98 (60, 136)		0 (0, 83)	

Abbreviations: ER, estrogen receptor; PgR, progesterone receptor; HER2, human epidermal growth factor receptor 2; AR, androgen receptor, NS, not significant

^a^ HR(+): estrogen receptor (+) and/or progesterone receptor (+)

* Factor showing statistical significance.

^$^ Kruskal-Wallis test

### Prognostic relevance of PA1 nuclear and cytoplasmic protein expression

In the analysis of RFS, local recurrences and distant metastases were considered as an event. 38 (11.0%) of breast cancer relapse, and 304 (88.3%) were relapse-free at the last follow-up. Two patients died of other cancer (renal cell carcinoma and primary unknown cancer). Out of 38 recurrent cases, 19 recurred from distant metastases, 7 from locally, and 12 showed both local and distance simultaneously. A total of 23 cases died from breast cancer, which was regarded as events when analyzing BCSS.

**Figure 2:**
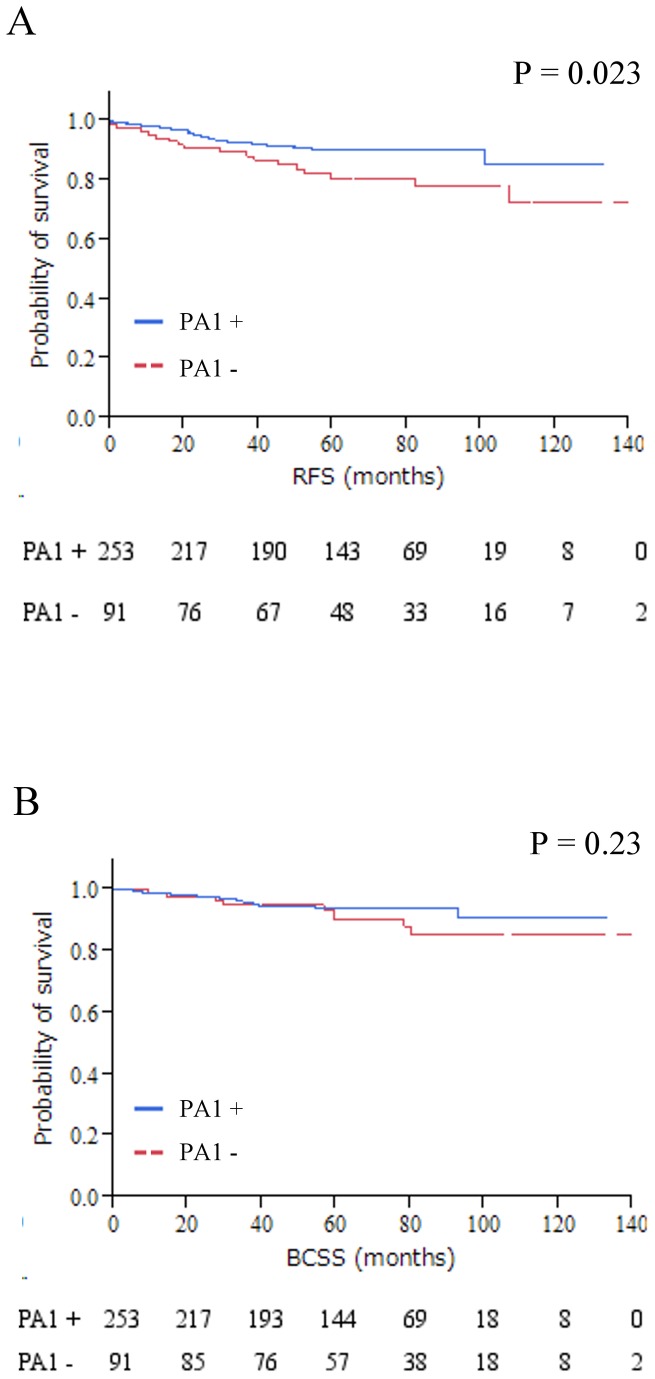
Kaplan–Meier plots of the association of PA1 expression in the nucleus with RFS; A and BCSS; B in the entire cohort. When PA1 protein expression was defined as either positive or negative, positive cases seemed to have a longer survival than negative ones in Kaplan–Meier method analysis (RFS, P = 0.023), but no slightly correlation with BCSS (P = 0.23). No association could be found between PA1 protein expression in the cytoplasm and RFS, BCSS (data not shown). Abbreviations: RFS, relapse-free survival; BCSS, breast cancer-specific survival.

Using the HS 25^th^ percentile as the cutoff point, patients showing higher PA1 expression in the nucleus were associated with prolonged RFS (P = 0.023) but not with BCSS (P = 0. 23) which was tested by Kaplan–Meier method and verified by the log-rank (Mantel–Cox) test (Figure 2).

### Univariate and Multivariate Prognostic Analysis of PA1 Protein Expression in Breast Cancer for RFS and BCSS

In the Cox hazards model, PA1-nuc protein expression proved to be a significant prognostic univariate parameter for RFS (Table 2, P = 0.03), but not for BCSS (Table 3, P = 0.25). There was no significant multivariate parameter for both RFS (Table 2, P = 0.20) and BCSS (Table 3, P = 0.63) in PA1-cyto protein positive group.

**Table 2:** Univariate and multivariate analysis for relapse-free survival (Cox proportional hazards model).

Variable		Value	n	Univariate analysis			Multivariate analysis		
				HR	95% CI	P Value	HR	95% CI	P Value
Age at operation	(ref = ≤ 50)	50 <	344	0.48	0.25-0.92	0.028*	0.50	0.24-1.09	NS
Menopause state	(ref = Pre)	Post	342	0.59	0.31-1.14	NS			
T stage	(ref = T1)	T2 <	343	2.30	1.20-4.80	0.0085*	1.36	0.60-3.2	NS
Node metastasis	(ref = 0)	Positive	330	2.30	1.20-4.50	0.011*	2.38	1.09-5.4	0.028
Histopathology	(ref = Lobular)	Ductal	312	0.49	0.14-3.06	NS			
Nuclear Grade	(ref = 1,2)	3	340	3.90	2.00-7.40	<0.0001*	2.5	1.07-6.08	0.034*
ERα (IHC)	(ref = <1%)	1% <	344	0.28	0.14-0.54	0.0003*	0.35	0.13-0.93	0.036*
PgR (IHC)	(ref = <1%)	1% <	342	0.37	0.19-0.71	0.0029*			
HER2	(ref = Negative)	Positive	344	1.40	0.57-3.03	NS			
Ki67 (MIB1)	(ref = < 15%)	15 <	327	2.40	1.10-6.10	0.018*	1.65	0.65-5.05	NS
AR	(ref = <1%)	1% <	338	0.37	0.18-0.82	0.016*	0.64	0.26-1.63	NS
ERβ	(ref = the 25^th^ percentile)	Positive	295	0.75	0.37-1.60	NS			
PA1 in the nucleus	(ref = the 25^th^ percentile)	Positive	344	0.48	0.25-0.93	0.03*	0.61	0.29-1.32	NS
PA1 in the cytoplasm	(ref = the 25^th^ percentile)	Positive	344	0.85	0.44-1.60	NS			
Systemic therapy	(ref = No)	Yes	340	0.87	0.34-2.90	NS			

Abbreviations: ER, estrogen receptor; PgR, progesterone receptor; HER2, human epidermal growth factor receptor 2; AR, androgen receptor; HR, Hazard Ratio; CI, Confidence Interval; NS, Not significant.

Considering the co-effect of PA1 expression with each factor was used in the multivariate analysis, respectively.

* Factor showing statistical significance;

**Table 3:** Univariate and multivariate analysis for breast cancer-specific survival (Cox proportional hazards model).

Variable		Value	n	Univariate analysis			Multivariate analysis		
				HR	95% CI	P Value	HR	95% CI	P Value
Age at operation	(ref = < 50)	50 <	344	1.54	0.61-4.60	NS			NS
Menopause state	(ref = Pre)	Post	342	1.50	0.60-4.50	NS			NS
T stage	(ref = T1)	T2 <	343	2.80	1.20-7.40	0.014*	1.21	0.42-3.79	NS
Node metastasis	(ref = 0)	Positive	330	3.20	1.30-8.10	0.0065*	3.92	1.44-11.8	0.0067
Histopathology	(ref = Lobular)	Ductal	312	0.25	0.07-1.60	NS			NS
Nuclear Grade	(ref = 1,2)	3	340	4.60	2.00-11.0	0.0004*	1.49	0.53-4.33	NS
ERα (IHC)	(ref = <1%)	1% <	344	0.12	0.05-0.29	<0.0001*	0.17	0.05-0.56	0.0032*
PgR (IHC)	(ref = <1%)	1% <	342	0.14	0.05-0.33	<0.0001*			
HER2	(ref = Negative)	Positive	344	0.58	0.09-2.00	NS			NS
Ki67 (MIB1)	(ref = < 15%)	15 <	327	6.00	1.70-37.5	0.0021*	3.30	0.91-21.1	NS
AR	(ref = <1%)	1% <	338	0.17	0.07-0.41	0.0002*	0.40	0.15-1.07	NS
ERβ	(ref = the 25^th^ percentile)	Positive	295	0.61	0.25-1.50	NS			NS
PA1 in the nucleus	(ref = the 25^th^ percentile)	Positive	344	0.60	0.26-1.40	NS			NS
PA1 in the cytoplasm	(ref = the 25^th^ percentile)	Positive	344	0.91	0.39-2.00	NS			NS
Systemic therapy	(ref = No)	Yes	340	0.22	0.18-1.60	NS			NS

Abbreviations: ER, estrogen receptor; PgR, progesterone receptor; HER2, human epidermal growth factor receptor 2; AR, androgen receptor; HR, Hazard Ratio; CI, Confidence Interval; NS, Not significant.

Considering the co-effect of PA1 expression with each factor was used in the multivariate analysis, respectively

* Factor showing statistical significance.

To further explore the prognostic value of PA1 in subgroups of breast cancer patients stratified by lymphnode or ER status. We found that there was a marginal significance for RFS in ER-negative (ER-) group with higher PA1-nuc level (Figure S1 B, P = 0.056). In addition, for those patients without lymphnode metastasis, PA1-nuc was a significant independent prognostic factor for RFS in both log-lank test (Figure S1 B, P = 0.025) and univariate and multivariate analyses (Table 4, P = 0.0374 and 0.045, respectively). 

**Table 4:** Univariate and multivariate analysis for relapse-free survival in node metastasis-free patients (Cox proportional hazards model).

Variable		Value	n	Univariate analysis			Multivariate analysis		
				HR	95% CI	P Value	HR	95% CI	P Value
Age at operation	(ref = < 50)	50 <	344	0.41	0.15-1.11	0.08			
Menopause state	(ref = Pre)	Post	342	0.67	0.24-1.97	0.44			
T stage	(ref = T1)	T2 <	343	2.02	0.74-5.52	0.16			
Histopathology	(ref = Lobular)	Ductal	312	0.26	0.05-4.95	0.29			
Nuclear Grade	(ref = 1,2)	3	340	6.04	2.16-17.2	0.0009*	5.50	1.96-15.7	0.0016*
ERα (IHC)	(ref = <1%)	1% <	344	0.49	0.18-1.59	0.22			
PgR (IHC)	(ref = <1%)	1% <	342	0.52	0.19-1.48	0.21			
HER2	(ref = Negative)	Positive	344	1.41	0.32-4.41	0.60			
Ki67 (MIB1)	(ref = < 15%)	15 <	327	2.24	0.76-8.13	0.14			
AR	(ref = <1%)	1% <	338	0.44	0.15-1.61	0.19			
ERβ	(ref = the 25^th^ percentile)	Positive	295	0.70	0.23-2.39	0.55			
PA1 in the nucleus	(ref = the 25^th^ percentile)	Positive	344	0.34	0.12-0.93	0.0374*	0.34	0.12-0.97	0.045*
PA1 in the cytoplasm	(ref = the 25^th^ percentile)	Positive	344	0.82	0.29-2.21	0.69			
Systemic therapy	(ref = No)	Yes	340	0.94	0.25-6.06	0.93			

Abbreviations: ER, estrogen receptor; PgR, progesterone receptor; HER2, human epidermal growth factor receptor 2; AR, androgen receptor; HR, Hazard Ratio; CI, Confidence Interval; NS, Not significant.

Considering the co-effect of PA1 expression with each factor was used in the multivariate analysis, respectively

* Factor showing statistical significance.

### PA1 Expression and Recurrence in ER-positive (ER+)/HER2-negative (HER2-) breast cancer

**Figure 3:**
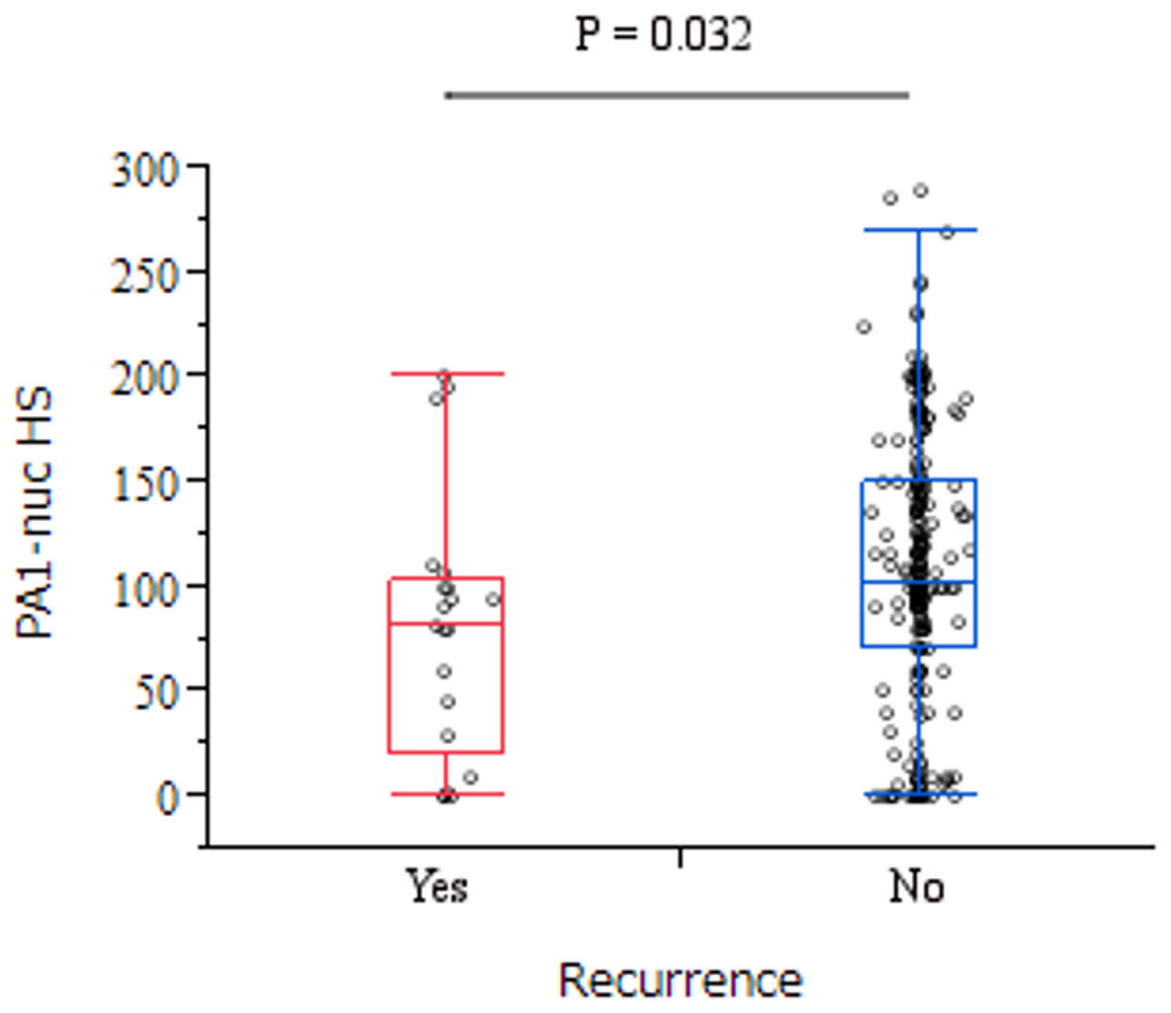
Box plots of the PA1-nuc H-score w/ vs. w/o breast cancer relapse in luminal A type breast cancer. There was significant difference between relapse group (median [25%, 75%]; 82 [20,103]) and no relapse one (101 [70,150]) in the Mann Whitney U test (P = 0.032). Abbreviations: PA1-nuc, nuclear PA1 expression; PA1-cyto, cytoplasmic PA1 expression.

In this study, the number of patients with recurrence was 21 (8.0 %) of 264 ER+/HER2- patients. There was a significant higher levels of PA1-nuc in the patients without recurrence than with (Figure 3, P = 0.032), but there was no significant difference in PA1-cyto w/o recurrence (data not shown, P = 0.24).

### Spearman rank correlation analysis between PA1-nuc/-cyto expression and nuclear receptor in the entire cohort

Previous PA1 nuclear receptor functional studies showed PA1 is a transactivator for ER, whereas a suppressor for GR [16,25]. It is of interests to enquire the correlation of PA1 protein with other members of nuclear receptor superfamily from the clinical samples. Using continuous data, the results of PA1-nuc and -cyto protein expression were compared with the breast hormone receptors in our entire cohort (Table S1). PA1-nuc and -cyto protein expression were significantly correlated with ERα (PA1-nuc; γ = 0.12, P = 0.02, PA1-cyto; γ = 0.13, P = 0.01) and ERβ (PA1-nuc; γ = 0.22, P < 0.0001, PA1-cyto; γ = 0.12, P = 0.03), however, there was no significant correlation with PgR in both nuclear and cytoplasmic cellular compartment of PA1.

## Discussion

PA1 was a newly identified PTIP associated protein [14] and was functionally involved in histone methyltransferase activity in epigenetic modulation of H3K4 in a situation without DNA damage stress. PA1 with PTIP was also found to participate in DNA damage response via ring finer protein 8 (RNF8), E3 ubiquitin-protein ligase, dependent pathway and was required for cell survival after DNA damage [25]. Recently, it was discovered that PA1 modulates transcriptional activity of nuclear receptor in a receptor-specific manner, which demonstrated to be an ER activator, GR suppressor, and also plays a role in estrogen stimulating G1/S cell cycle progression [15,16]. However, the role of PA1 in breast cancer, of which growth and progression were highly related with hormone receptors, remains unknown. All these former interesting findings prompted us to investigate PA1 expression and reveal its function, using a large cohort of 344 cases of consecutive invasive breast cancer samples.

In the present study, PA1 expression level was assayed by immunohistochemistry and quantified by immunohistochemistry HS. Our results indicated that the positive PA1-nuc staining was significantly associated with postmenopausal group (P = 0.0097), smaller tumor size (P = 0.0025), negative Ki67 (P = 0.02), positive AR (P = 0.049), and positive ERβ (P = 0.0020) status. Using the HS 25^th^ percentile as the cutoff point, patients showing higher PA1-nuc were associated with prolonged RFS (P = 0.023) but not with BCSS (P = 0. 23) (Figure 2). Furthermore, the prognosis value of PA1-nuc protein was verified by Cox Hazardous model analysis, and it demonstrated that PA1-nuc protein expression is a prognostic univariate parameter for RFS, but not for BCSS. It suggested that PA1-nuc has some value to predict RFS, but is not an independent prognostic factor, which is affected by other clinicopathological factors such as tumor proliferation property Ki-67 level etc. BCSS is defined that only deaths from the disease of breast cancer are counted. PA1-nuc protein expression is a prognostic univariate parameter for RFS, not for BCSS might suggest that the tumor suppressor like activity of PA1 is limited before any kind of relapse occurs. It is very interesting to find the positive effect of PA1-nuc correlated with better survival and non-malignant tumor characteristics and function like a tumor suppressor. However, the underlying mechanism remains unclear and further functional studies are warranted. Further analyses for those patients without lymphnode negative metastasis, PA1-nuc positive patients showed significant extended RFS and BCSS and the prognostic value was convinced by univariate and multivariate analyses. Spearman rank correlation analysis revealed that either PA1-nuc or -cyto protein expression was correlated with ERα (γ = 0.12, P = 0.02) and ERβ (γ = 0.22, P < 0.0001). However, there was no significant correlation with PgR in both nuclear and cytoplasmic cellular compartment of PA1, which may be caused by receptor-specific modulation on its transactivity. Particularly, the highly significant correlation between PA1 and ERβ suggested that PA1 might be able to interact and modulate ERβ transcriptional activity in breast cancer. The highly significant correlation of PA1-nuc/ -cyto with ERα, a well-established/widely accepted endocrine therapy responsiveness index, might suggest that PA1 is of potential value to be a candidate for endocrine therapy responsive indicator and therefore increase the accuracy of prediction in combination with ERα, which warrants further confirmation in a well-defined cohort of endocrine treated breast cancer patients. Higher levels of PA1-nuc protein in 243 relapse-free patients, compared with 21 relapsed patients, and the significant PA1 correlation with non-aggressive clinicopathological parameters, may suggest that PA1 has tumor-suppressor-like activity and functions as a candidate tumor suppressor in ER+/HER2- subtype. 

We observed pure PA1-nuc presents in 145 (42.1%), pure PA1-cyto in 9 (2.6%), and both PA1-nuc/-cyto in 168 (48.8%) of 344 cases, respectively. The underlying mechanism of different staining patterns as shown in the present study remains unknown. It may suggest protein functional difference of PA1 protein in different subcellular compartments. The cytoplasmic existing of PA1 protein may suggest different functional role of PA1 other than DNA repair [25] or nuclear receptor transactivity modulation [15,16], which are occurring mainly in the nuclear compartment. The shuttling mechanism of PA1 protein between nuclear and cytoplasm is largely unknown. It is also of importance to investigate the naturally occurring PA1 mutations that may affect PA1 expression and function in human cancer. The significant positive correlation between PA1 and ERs or AR, strongly suggested that further investigational study on PA1 expression in the other hormone related cancers such as prostate cancer, ovarian cancer and endometrial cancer. Furthermore, it would be interesting to examine PA1 expression in a certain type of cancer, which seems to be related with hormone receptor but not fully related, like lung cancer [26] or hepatocellular carcinoma [27] to find how PA1 is associated with initiation and growth for these types of cancers. 

In conclusion, PA1 nuclear expression was found to be an independent RFS prognostic indicator for those breast cancer patients without lymphnode metastasis. 

## Supporting Information

Figure S1 **Kaplan-Meier plots illustrating the RFS of patients by ER-positive; A, ER-negative; B and node metastases-positive; C, node metastases-negative; D.** PA1 cutoff was the same as for Figure 2. Abbreviation: RFS, relapse-free survival.
(TIF)

Table S1 **The correlation between PA1 nuclear/cytoplasmic protein expression and each steroid receptor in the entire cohort, using Spearman rank correlation test.**
(DOCX)
