# Supplementary figures and images for: PTIP Associated protein 1, PA1, Is an Independent Prognostic Factor for Lymphnode Negative Breast Cancer
Source: PLoS One. 2013 Nov 18;8(11):e80552. doi: 10.1371/journal.pone.0080552 (PMC3832393; doi:10.1371/journal.pone.0080552)

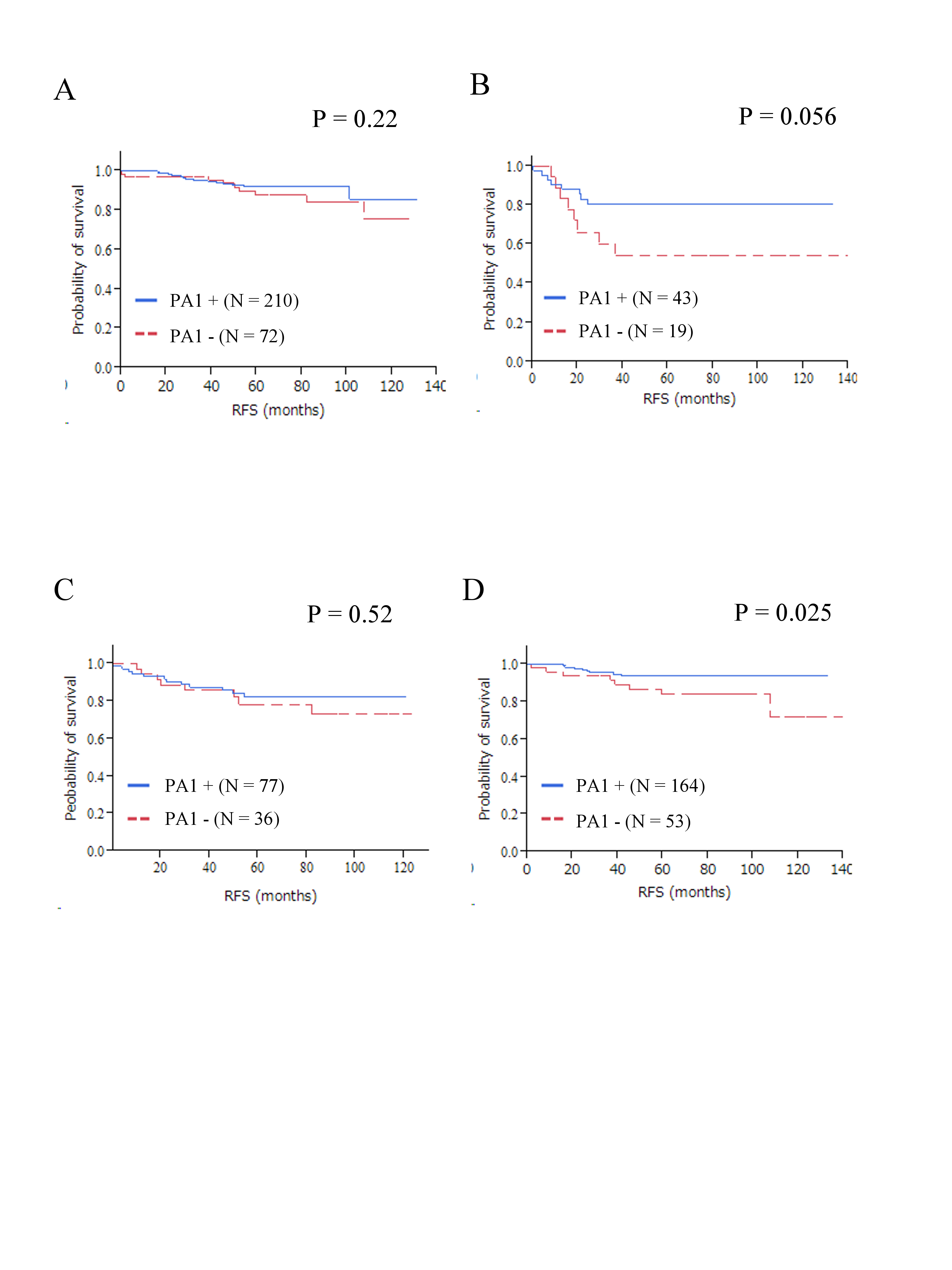

Supplement: Figure S1 — Kaplan-Meier plots illustrating the RFS of patients by ER-positive; A, ER-negative; B and node metastases-positive; C, node metastases-negative; D. PA1 cutoff was the same as for Figure 2. Abbreviation: RFS, relapse-free survival. (TIF) [file pone.0080552.s001.tif]
