# Supplementary material for: PTIP Associated protein 1, PA1, Is an Independent Prognostic Factor for Lymphnode Negative Breast Cancer
Source: PLoS One. 2013 Nov 18;8(11):e80552. doi: 10.1371/journal.pone.0080552 (PMC3832393; doi:10.1371/journal.pone.0080552)
Supplement: Table S1 — The correlation between PA1 nuclear/cytoplasmic protein expression and each steroid receptor in the entire cohort, using Spearman rank correlation test. (DOCX) [file pone.0080552.s002.docx]

*Supplementary Table S1* The correlation between PA1 nuclear/cytoplasmic protein expression and each steroid receptor in the entire cohort, using Spearman rank correlation test.

| parameter | PA1 (HS) | | | |
| --- | --- | --- | --- | --- |
|  | Nucleus | | Cytoplasm | |
|  | γ | p-value | γ | p-value |
| ERα (%) | 0.1240 | 0.0214* | 0.1340 | 0.0128* |
| PR (%) | 0.0274 | NS | 0.0125 | NS |
| AR (HS) | 0.1359 | 0.0124* | 0.0815 | NS |
| ERβ (HS) | 0.2250 | <0.0001* | 0.1253 | 0.0314* |

Abbreviations: ER, estrogen receptor; PgR, progesterone receptor; AR, androgen receptor.

* Factor showing statistical significance.
